# Adaptive control of time delay teleoperation system with uncertain dynamics

**DOI:** 10.3389/fnbot.2022.928863

**Published:** 2022-07-22

**Authors:** Siyu Lu, Yuxi Ban, Xia Zhang, Bo Yang, Shan Liu, Lirong Yin, Wenfeng Zheng

**Affiliations:** ^1^School of Automation, University of Electronic Science and Technology of China, Chengdu, China; ^2^Department of Geography and Anthropology, Louisiana State University, Baton Rouge, LA, United States

**Keywords:** adaptive bilateral control method, Lyapunov stability theory, time delay teleoperation system, uncertain dynamics, force feedback, PEB control structure

## Abstract

A bilateral adaptive control method based on PEB control structure is designed for a class of time-delay force feedback teleoperation system without external interference and internal friction to study the uncertainty of dynamic parameters and time delay. The stability and tracking performances of the closed-loop constant time delay teleoperation system are analyzed by Lyapunov stability theory. Finally, the controller designed in this paper is successfully applied to the teleoperation system composed of a two-degree of freedom rotating manipulator as the master robot and the slave robot. The simulation is carried out in no operator and environment force or with operator and environment force. The adaptive bilateral control method's control performance is compared with that of the traditional time-delay teleoperation system. Finally, it is verified that the method has good control performance.

## Introduction

After decades of research and rapid development of teleoperation robot systems, they have been applied in many fields, such as unmanned submersibles (Sayers and Paul, 1994), space robots (Bejczy, 1994), remote surgery robots (Wright et al., 2006; Su et al., 2020c; Tang et al., 2020), and teleoperation mobile robots (DiMaio et al., 2011; Su et al., 2020a,b,d). In a word, through the research of teleoperation robot systems, human intelligence and robotics can be combined to improve efficiency and reduce cost when performing tasks extensively. Therefore, it has a broad application prospect and rich practical significance.

Usually, in a teleoperation robot system, the operator controls the equipment at the main end to carry out a particular action and then transmits the equipment's action signal at the main end to the controller of the slave end through the communication channel. It then predicts and post-processes the device's control signal at the slave end by calculation (Zhang et al., 2022; Zheng et al., 2022), commands the equipment at the slave end, realizes the human's work skills, and completes the corresponding work tasks. The available remote operation robot system includes a master module, operator module, and the Master Controller, communication channel, Slave Controller, Slave, and environment. Cho and Park (2005) designed an impedance controller for teleoperation systems with communication delay for varying damping. Do and Namerikawa (2009) proposed an impedance control method for the time-delay force feedback teleoperation system based on IOS small gain theory. However, the transparency of the teleoperation system with impedance control structure is not ideal.

Position error-based structure (PEB), is a control structure that uses the position signals of the master robot and the slave robot as its own desired signals, and then designs a controller for the generated error signals so as to achieve the goal of system stability and improve tracking performance. When the slave robot contacts the working environment in the teleoperation system, the weighted position error can be fed back to the operator so that the position error presents the interaction force, and then the control torque can be adjusted to reduce the tracking error between the master robot and the slave robot. Nuño et al. (2008) designed a globally stable PD bilateral controller based on the bilateral teleoperation system position error structure. Nuño et al. (2008) proposed an adaptive bilateral control method based on the non-linear teleoperation system's position error structure. Joinié-Maurin et al. (2011) proposed a force feedback teleoperation system based on position error structure. They can compensate for external interference of the system and solve the influence of interference on system stability. Franken et al. (2012) proposed a stable position-based teleoperation system by introducing damping compensation. Kim and Ahn (2013) used a composite adaptive controller to design a teleoperation system based on position differences. Lawrence (1994), Yokokohji and Yoshikawa (1994), and Hastrudi-Zaad and Salcudean (1999) proposed a remote operation system based on four communication channels (4-Channel, 4-Ch). The teleoperation system has been proved to have good force tracking and transparency. Liu and Tavakoli (2011) proposed an adaptive inverse dynamics control method for a non-linear uncertain teleoperation system based on a 4-Ch structure. Dehghan et al. (2016) proposed a non-linear bilateral control method based on a 4-Ch teleoperation system using an adaptive force estimator.

Most of the teleoperation systems studied are based on the position-position type and 4-Ch control structure because these two structures have better characteristics.

The control signals between the master robot and the slave robot communicate through the forward communication channel and the reverse communication channel. Usually, the distance between the system's communication network channels is relatively long, which will cause delays. As should go without saying, the existence of a time delay will lead to 'poor system performance. It will bring the problem of destroying the stability of the teleoperation system. The master-slave robot's dynamic mathematical model is established in the teleoperation system by analyzing its motion characteristics. The structure and mechanical parameters of the master and slave robots are involved in the mathematical model (Liu et al., 2021; Zhang et al., 2022). In general, most of the teleoperation system's control methods are realized under the condition of the master-slave robot's precise structure and mechanical parameters. However, in the actual teleoperation mechanical system, it is difficult to obtain the robot's precise mechanical parameters, such as mass, length, the center of mass, and moment of inertia. As a result, the system dynamic parameters (inertia vector matrix, centrifugal force matrix, and gravity term matrix) are not accurate, which is common in robot workspace control. In this case, it is impossible to establish an accurate mathematical model of the teleoperation system, which will reduce the system's transparency and performance and even cause the whole system instability. After the above analysis, considering the influence of dynamic parameter uncertainty and time delay, we mainly solve the two problems of time delay and dynamic parameter uncertainty in the teleoperation system.

For the above two problems to be solved in this paper, from the mentioned teleoperation system's research status, we can conclude that there are mainly adaptive control methods to solve communication channel delay and system dynamic parameter uncertainty (Polushin et al., 2010; Haddadi et al., 2015; Wang et al., 2021).

Moreover, most of the existing bilateral adaptive control methods for time-delay teleoperation systems do not consider operator and environmental dynamic parameter uncertainty. They usually choose the adaptive law proposed by Slotine and Li (1991) to estimate the system dynamic parameter vector. However, Slotine and Li (1991) also pointed out that if the reference signal does not meet the persistent excitation condition, the estimated value's accuracy cannot be guaranteed.

Based on the above discussion, in this paper, based on the position error control structure, a new adaptive bilateral teleoperation control method is designed. This new method solves the problems of constant time delay and uncertain dynamic parameters in the teleoperation system. It will ensure the closed-loop constant time-delay system's stability and improve the system's tracking control (Xu et al., 2020; Zhang et al., 2021) performance.

## Method and experiments

### Mathematical model of teleoperation system

#### The spatial dynamic model of master-slave robot joint

Without considering the joint friction and external interference, the general dynamic equations of the master robot and the slave robot of the teleoperation system can be expressed by the following Euler Lagrange formula (Nuño et al., 2008):


(1) $$\begin{array}{l} M_{q_{m}}\left(q_{m}\right){\ddot{q}}_{m}+C_{q_{m}}\left(q_{m},{\dot{q}}_{m}\right){\dot{q}}_{m}+G_{q_{m}}\left(q_{m}\right)=\tau_{m}+J_{m}^{T}\left(q_{m}\right)F_{h} \end{array}$$

(2) $$\begin{array}{l} M_{q_{s}}\left(q_{s}\right){\ddot{q}}_{s}+C_{q_{s}}\left(q_{s},{\dot{q}}_{s}\right){\dot{q}}_{s}+G_{q_{s}}\left(q_{s}\right)=\tau_{s}-J_{s}^{T}\left(q_{s}\right)F_{e} \end{array}$$

In order to simplify the description, the subscripts *i*(*i* = *m, s*) are defined to represent the master and slave robots. Then $q_{i}\in {ℜ}^{n\times 1}$, ${\dot{q}}_{i}\in {ℜ}^{n\times 1}$, and ${\ddot{q}}_{i}\in {ℜ}^{n\times 1}$ are divided into the joint angular position, angular velocity, and angular acceleration of the master robot and the slave robot. $M_{q_{i}}\left(q_{i}\right)\in {ℜ}^{n\times n}$ is the inertia matrix of the master robot and the slave robot. $C_{q_{i}}\left(q_{i},{\dot{q}}_{i}\right)\in {ℜ}^{n\times n}$ is the Coriolis force and centripetal force matrix of the master robot and the slave robot. $\tau_{i}\in {ℜ}^{n\times 1}$ is the joint control torque of the master robot and the slave robot. $G_{q_{i}}\left(q_{i}\right)\in {ℜ}^{n\times 1}$ is the gravity matrix of the slave robot. $J_{i}\left(q_{i}\right)\in {ℜ}^{n\times n}$ represents the Jacobian matrix of the master robot and the slave robot. $J_{i}^{T}\left(q_{i}\right)$ is the transposition of the Jacobian matrix. $F_{h}\in {ℜ}^{n\times 1}$ represents the force exerted by the operator on the master robot and $F_{e}\in {ℜ}^{n\times 1}$ is the interaction force between the slave robot and the environment module.

The dynamic equations of the master robot and slave robot in the teleoperation system, namely formula (1) and formula (2), have the following properties:

Property (1): the inertia matrix, *M*_*q*_*i*__(*q*_*i*_), is symmetric and positive definite, with maximum and minimum values.


(3) $$\begin{array}{l} 0<\lambda_{\min}{\left\{M_{q_{i}}\left(q_{i}\right)\right\}I\leq M}_{q_{i}}\left(q_{i}\right)\leq \lambda_{\max}\left\{M_{q_{i}}\left(q_{i}\right)\right\}I<\infty \end{array}$$

Property (2): the matrix of Coriolis and centrifugal force $C_{q_{i}}\left(q_{i},{\dot{q}}_{i}\right)$ and satisfies: ${Ṁ}_{q_{i}}\left(q_{i}\right)-2C_{q_{i}}\left(q_{i},{\dot{q}}_{i}\right)$ is skew-symmetric. Namely:


(4) $$\begin{array}{l} \zeta^{T}\left({Ṁ}_{q_{i}}\left(q_{i}\right)-2C_{q_{i}}\left(q_{i},{\dot{q}}_{i}\right)\right)\zeta =0,\forall \zeta \in {ℜ}^{n\times 1} \end{array}$$

Equivalently, we can get $\zeta^{T}\left(\frac{1}{2}{Ṁ}_{q_{i}}\left(q_{i}\right)-C_{q_{i}}\left(q_{i},{\dot{q}}_{i}\right)\right)\zeta =0,\forall \zeta \in {ℜ}^{n\times \;1}$

Property (3): We linearly transform the terms on the left of the dynamic formulas (1) and (2) of the master robot and the slave robot of the teleoperation system, and define the unknown constant parameter vector of the robot as $\theta_{d}={\left[\theta_{d1},\cdot \cdot \cdot ,\theta_{dr}\right]}^{T}$, then θ_*d*_ is linear, and:


(5) $$\begin{array}{l} M_{q_{i}}\left(q_{i}\right){\ddot{q}}_{i}+C_{q_{i}}\left(q_{i},{\dot{q}}_{i}\right){\dot{q}}_{i}+G_{q_{i}}\left(q_{i}\right)=\tau_{i}=Y_{d}\left(q_{i},{\dot{q}}_{i},{\ddot{q}}_{i}\right)\theta_{d} \end{array}$$

Among them, $Y_{d}\left(q_{i},{\dot{q}}_{i},{\ddot{q}}_{i}\right)\in {ℜ}^{n\times r}$ is called the dynamic regression matrix, which is the known function matrix about the robot's joint variables.

#### Kinematics model of master-slave robot in workspace

Through geometric analysis of the structure of the robot, we can establish that there is a conversion relationship between the joint position of the robot and the end position of the robot actuator $x_{m},x_{s}\in {ℜ}^{n\times 1}$ can be expressed as


(6) $$\begin{array}{l} x_{m}=h_{m}\left(q_{m}\right),x_{s}=h_{s}\left(q_{s}\right) \end{array}$$

Among them, $h_{i}\left(q_{i}\right)\in {ℜ}^{n}\to {ℜ}^{n}$ is a non-linear transformation used to describe the relationship between the master-slave robot actuator's end position and the joint space angle position. Thus, the end velocity of the actuator in the workspace of the master robot and the slave robot is:


(7) $$\begin{array}{l} {ẋ}_{m}=J_{m}\left(q_{m}\right){\dot{q}}_{m},{ẋ}_{s}=J_{s}\left(q_{s}\right){\dot{q}}_{s} \end{array}$$

Among them, *J*_*m*_(*q*_*m*_), *J*_*s*_(*q*_*s*_) are the Jacobian matrix of the master robot and the slave robot, respectively. For the sake of simplicity, it is abbreviated as *J*_*m*_, *J*_*s*_.

Derivation of formula (7) to time, the acceleration of the executive end of the master-slave robot in the workspace is obtained as follows:


(8) $$\begin{array}{l} {ẍ}_{m}={\dot{J}}_{m}{\dot{q}}_{m}+J_{m}{\ddot{q}}_{m} \end{array}$$

(9) $$\begin{array}{l} {ẍ}_{s}={\dot{J}}_{s}{\dot{q}}_{s}+J_{s}{\ddot{q}}_{s} \end{array}$$

Therefore, the above formula's joint space models (1) and (2) can be transformed into the workspace's motion model. It can better describe the robot's contact and the environment and more intuitively relate the robot's end-effector's velocity and acceleration with force acting on the environment's end.

#### The dynamic model of operator and environment workspace

The operator and environment dynamic models in formulas (1) and (2) of the teleoperation system are usually described by the workspace robot's end actuator position. The expressions of the force exerted by the operator of the master robot's actuator and the interaction between the slave robot and the environment are as follows:


(10) $$\begin{array}{l} F_{h}=f_{h}^{{\;}^{*}}-\Phi_{h}\left(x_{m},{ẋ}_{m},{ẍ}_{m}\right) \end{array}$$

(11) $$\begin{array}{l} F_{e}=f_{e}^{{\;}^{*}}+\Phi_{e}\left(x_{s},{ẋ}_{s},{ẍ}_{s}\right) \end{array}$$

Among them, $f_{h}^{*}$ represents the external force exerted by the operator on the main robot, meeting the requirements $\|f_{h}^{*}{\|}_{\infty }\leq \alpha_{h}$. $f_{e}^{*}$ represents the external force applied by the working environment module to the slave robot. Φ_*h*_(*x*_*m*_, ẋ_*m*_, ẍ_*m*_) and Φ_*e*_(*x*_*s*_, ẋ_*s*_, ẍ_*s*_) represent the operator's inertial and viscous characteristics and the working environment model, respectively, and can be linear or non-linear passive dynamic functions.

The inertia and viscosity functions in the dynamic model of the operator and environment workspace are represented as Φ_*h*/*e*_(*x*_*i*_, ẋ_*i*_, ẍ_*i*_), *i* = *m, s*. According to the literature (Malysz and Sirouspour, 2009; Haddadi et al., 2015), Φ_*h*/*e*_(*x*_*i*_, ẋ_*i*_, ẍ_*i*_) can include the following three situations:

Non-linear model: Φ_*h*/*e*_(*x*_*i*_, ẋ_*i*_, ẍ_*i*_) = *I*_*h*/*e*_(*x*_*i*_)ẍ_*i*_ + *H*_*h*/*e*_(*x*_*i*_, ẋ_*i*_) where *I*_*h*/*e*_(*x*_*i*_) and *H*_*h*/*e*_(*x*_*i*_, ẋ_*i*_) are differentiable function parameters.

The formula of non-linear viscous function combined with linear damping: $\Phi_{h/e}\left(x_{i},{ẋ}_{i},{ẍ}_{i}\right)={\bar{\Phi }}_{h/e}\left(x_{i},{ẋ}_{i}\right)+B_{h/e}\left(t\;\right){ẋ}_{i}$;

A second-order decoupled linear-time-invariant (LTI) model is formulated as: Φ_*h*/*e*_(*x*_*i*_, ẋ_*i*_, ẍ_*i*_) = *M*_*h*/*e*_ ẍ_*i*_ + *B*_*h*/*e*_ ẋ_*i*_ + *K*_*h*/*e*_*x*_*i*_ where $M_{h},B_{h},K_{h},M_{e},B_{e},K_{e}\in {ℜ}^{n\times n}$ is a positive definite constant diagonal matrix corresponding to the mass, damping, and elastic coefficient matrices of the operator and the environment.

For different application scenarios, the dynamic models of the operator and environment workspace are different. Because the inertia and viscosity characteristics of the workspace dynamic model of the operator and the environment are very complex, this paper selects the second-order decoupling linear-time-invariant (LTI) model in (3) to approximately establish the dynamic model of the operator and environment workspace in the teleoperation system.

To sum up, we can observe the dynamic model of the operator and environment workspace in the teleoperation system. In practical application, the teleoperation system's dynamic parameters' uncertainty includes master and slave robots' mechanical parameters and the operator and environment models' mechanical parameters, such as mass coefficient, damping coefficient, and elastic coefficient.

#### Joint space dynamic model of combined teleoperation system

By establishing the master robot module's mathematical models, the slave robot module, the operator module, and the environment module in the teleoperation system, we can observe the master and slave robots' dynamic models in the joint space. The dynamic model of the operator and the environment is in the working space. Thus, the teleoperation system's dynamic model cannot be unified, and the dynamic model is increased. It is challenging to design a bilateral controller. Therefore, it is necessary to use the master robot and slave-robot workspace kinematics model to transform the operator module's dynamic model and environment module workspace into joint space. Finally, the joint space dynamic model of the master robot and slave robot is sorted out. Then the simplified joint space model of the teleoperation system is obtained. By substituting formula (6) to formula (9) into formula (10) and formula (11), the joint space dynamic model of operator and environment is obtained as follows:


(12) $$\begin{array}{l} F_{h}=f_{h}^{{\;}^{*}}-M_{h}J_{m}{\ddot{q}}_{m}-\left(B_{h}J_{m}+M_{h}{\dot{J}}_{m}\right){\dot{q}}_{m}-K_{h}h\left(q_{m}\right) \end{array}$$

(13) $$\begin{array}{l} F_{e}=f_{e}^{{\;}^{*}}+M_{e}J_{s}{\ddot{q}}_{s}-\left(B_{e}J_{s}+M_{e}{\dot{J}}_{s}\right){\dot{q}}_{s}-K_{e}h\left(q_{s}\right) \end{array}$$

The two sides of formula (12) and formula (13) are multiplied by $J_{m}^{T}$ and substituting into formula (1) and formula (2), respectively, the simplified joint space model of the teleoperation system is obtained:


(14) $$\begin{array}{l} M_{m}\left(q_{m}\right){\ddot{q}}_{m}+C_{m}\left(q_{m},{\dot{q}}_{m}\right){\dot{q}}_{m}+G_{m}\left(q_{m}\right)=\tau_{m} \end{array}$$

(15) $$\begin{array}{l} M_{s}\left(q_{s}\right){\ddot{q}}_{s}+C_{s}\left(q_{s},{\dot{q}}_{s}\right){\dot{q}}_{s}+G_{s}\left(q_{s}\right)=\tau_{s} \end{array}$$

Among which:


(16) $$\begin{array}{l} M_{m}\left(q_{m}\right)=M_{q_{m}}\left(q_{m}\right)+{J_{m}}^{T}M_{h}J_{m} \end{array}$$

(17) $$\begin{array}{l} C_{m}\left(q_{m},{\dot{q}}_{m}\right)=C_{q_{m}}\left(q_{m},{\dot{q}}_{m}\right)+{J_{m}}^{T}B_{h}J_{m}+{J_{m}}^{T}M_{h}{\dot{J}}_{m} \end{array}$$

(18) $$\begin{array}{l} G_{m}\left(q_{m}\right)=G_{q_{m}}\left(q_{m}\right)+{J_{m}}^{T}K_{h}h_{m}\left(q_{m}\right)-{J_{m}}^{T}f_{h}^{{\;}^{*}} \end{array}$$

(19) $$\begin{array}{l} M_{s}\left(q_{s}\right)=M_{q_{s}}\left(q_{s}\right)+{J_{s}}^{T}M_{e}J_{s} \end{array}$$

(20) $$\begin{array}{l} C_{s}\left(q_{s},{\dot{q}}_{s}\right)=C_{q_{s}}\left(q_{s},{\dot{q}}_{s}\right)+{J_{s}}^{T}B_{e}J_{s}+{J_{s}}^{T}M_{e}{\dot{J}}_{s} \end{array}$$

(21) $$\begin{array}{l} G_{s}\left(q_{s}\right)=G_{q_{s}}\left(q_{s}\right)+{J_{s}}^{T}K_{e}h_{s}\left(q_{s}\right)+{J_{s}}^{T}f_{e}^{{\;}^{*}} \end{array}$$

After unifying the dynamics of each module in the teleoperation system into the joint space, according to property (1) to property (3), we can deduce the mathematical models of the combined teleoperation system described in formula (14) and formula (15) have the following new properties. for all *i* = *m, s* they, respectively represent the master and the slave.

Property (4): the inertial matrix *M*_*i*_(*q*_*i*_) is symmetric and positive definite, with maximum and minimum values.


(22) $$\begin{array}{l} 0<\lambda_{\min}{\left\{M_{i}\left(q_{i}\right)\right\}I\leq M}_{i}\left(q_{i}\right)\leq \lambda_{\max}\left\{M_{i}\left(q_{i}\right)\right\}I<\infty \end{array}$$

Property (5): For ∀ξ ∈ ℜ^*n*×1^, the Coriolis matrix Ṁ_*i*_(*q*_*i*_) and the centrifugal force matrix $C_{i}\left(q_{i},{\dot{q}}_{i}\right)$ satisfy:


(23) $$\begin{array}{l} \xi^{T}\left({Ṁ}_{i}\left(q_{i}\right)-2C_{i}\left(q_{i},{\dot{q}}_{i}\right)\right)\xi =-2\xi^{T}B_{i}\xi \end{array}$$

Property (6): unify each module's mathematical models in the teleoperation system into the joint space and substitute them into the joint space mathematical models of the master robot and the slave robot. After sorting out the teleoperation system's dynamic models, the items on the left of formula (14) and formula (15) are obtained. The unknown constant parameter vector of the robot can also be defined as $\theta_{z}={\left[\theta_{z1},\cdot \cdot \cdot ,\theta_{zr}\right]}^{T}$. After linear transformation, it is obtained that the parameter vector θ_*z*_ of the robot is linear.


(24) $$\begin{array}{l} M_{i}\left(q_{i}\right){\ddot{q}}_{i}+C_{i}\left(q_{i},{\dot{q}}_{i}\right){\dot{q}}_{i}+G_{i}\left(q_{i}\right)=\tau_{i}=Y_{z}\left(q_{i},{\dot{q}}_{i},{\ddot{q}}_{i}\right)\theta_{z} \end{array}$$

Where *i* = *m, s*, $Y_{z}\left(q_{i},{\dot{q}}_{i},{\ddot{q}}_{i}\right)\in {ℜ}^{n\times r}$ is called the dynamic regression matrix, which is the known function matrix about the robot's joint variables.

### Control structure based on the position error

**Figure 1:**
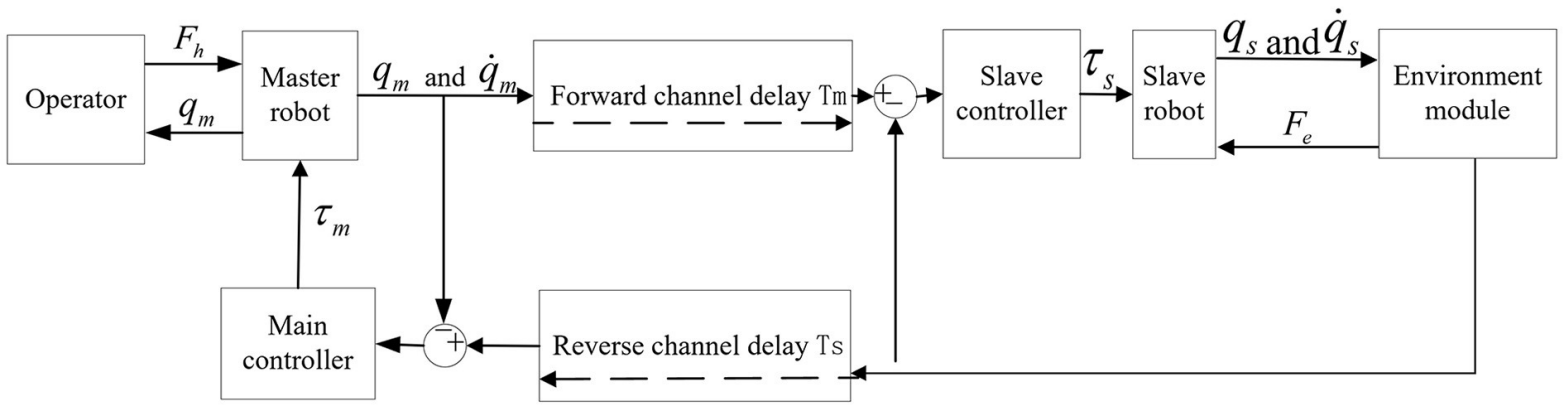
Control structure diagram of teleoperation robot system based on the position error.

The control structure of the teleoperation robot system based on position error (Bessa et al., 2010; Polushin et al., 2010) is shown in Figure 1. The control structure connects the master-slave robot's joint position and speed signals through communication channels to form a network. In this network, the master-slave robot's desired position and speed signals are the joint position and speed signals of the slave robot through the reverse channel. The expected position and speed signals of the slave robot are the joint position and speed signals of the master robot through the forward channel, meeting the following requirements:


(25) $$\begin{array}{l} \{\begin{array}{c} q_{s}^{d}\left(t\right)=q_{m}\left(t-T_{m}\right)\\ q_{m}^{d}\left(t\right)=q_{s}\left(t-T_{s}\right) \end{array},\;\{\begin{array}{c} {\dot{q}}_{s}^{d}\left(t\right)={\dot{q}}_{m}\left(t-T_{m}\right)\\ {\dot{q}}_{m}^{d}\left(t\right)={\dot{q}}_{s}\left(t-T_{s}\right) \end{array} \end{array}$$

Where $q_{m}^{d}\left(t\right)$,$q_{s}^{d}\left(t\right)$,${\dot{q}}_{s}^{d}\left(t\right)$,${\dot{q}}_{m}^{d}\left(t\right),$ respectively represent the expected position and speed signals of the master-slave robot. *q*_*m*_(*t* − *T*_*m*_), *q*_*s*_(*t* − *T*_*s*_), ${\dot{q}}_{m}\left(t-T_{m}\right)$, and ${\dot{q}}_{s}\left(t-T_{s}\right),$ respectively represent the position and speed signals of the master-slave robot joints passing through the communication channel, *T*_*m*_, *T*_*s*_, respectively represent the delay of the forward communication channel and the delay of the reverse communication channel.

As shown in Figure 1, this structure uses the position signals of the master robot and the slave robot as their expected signals. It then designs the controller for the generated error signals to achieve system stability and improve tracking performance. In the teleoperation system, when the slave robot and the working environment contact each other, the weighted position error can be fed back to the operator so that the interaction force can be presented through the position error. The control torque can then be adjusted to reduce the tracking error between the master robot and the slave robot. Therefore, the teleoperation system based on position error control structure is simple.

### The proposed teleoperation system with adaptive control

This paper is devoted to solving constant communication delay and dynamic parameter uncertainty in the teleoperation system. For a teleoperation system without considering the master-slave robot mechanism's internal friction and external interference, the joint space dynamic model can be expressed by formulas (14) and (15). An adaptive control method is designed. We apply the designed bilateral controller to the teleoperation system of the master robot and the slave robot. They are composed of two degrees of freedom and two link manipulators.

**Figure 2:**
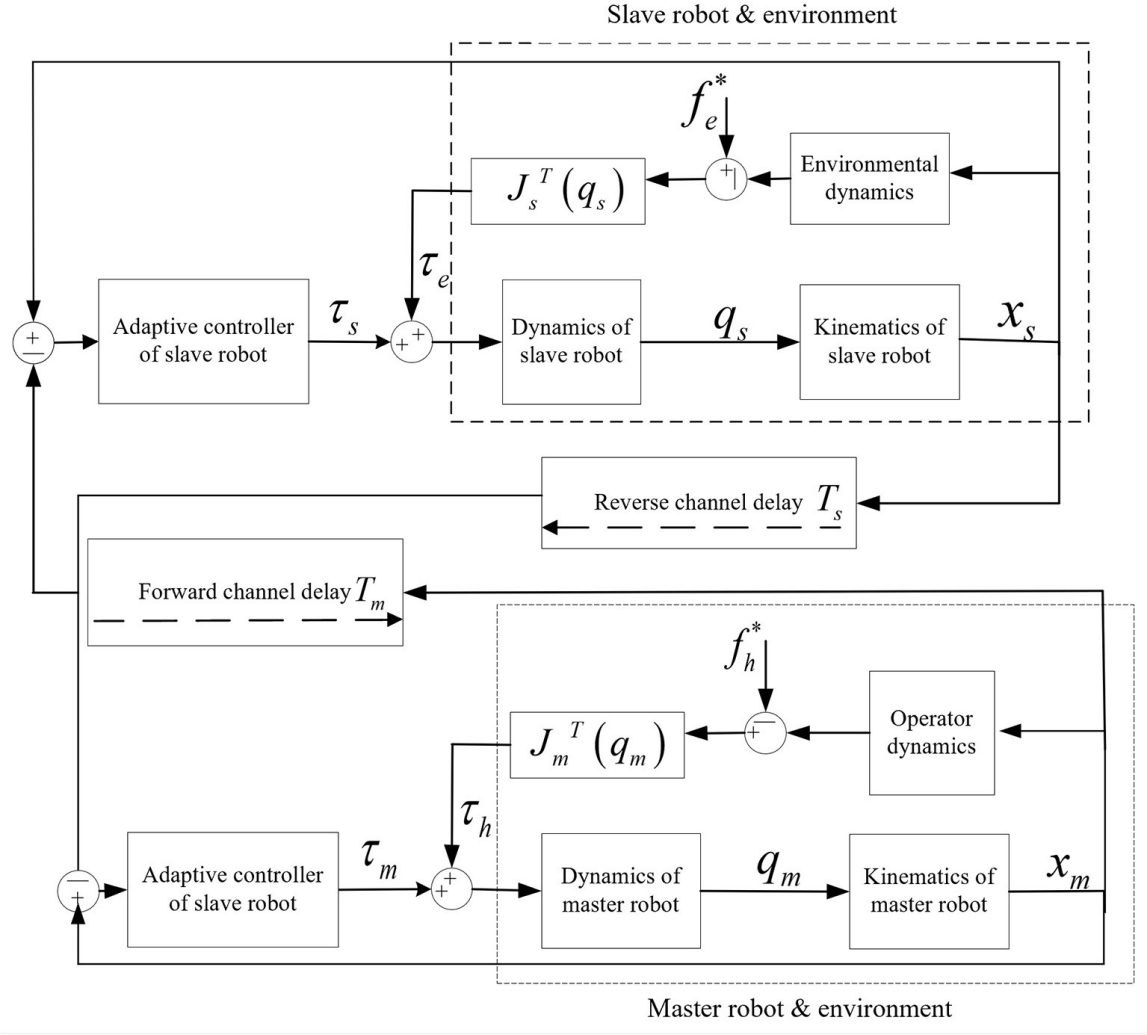
Adaptive control block diagram of teleoperation system based on position error structure.

In the combined teleoperation system introduced in Section Joint space dynamic model of combined teleoperation system, there is a constant communication delay between the forward channel and the reverse channel. Therefore, the combined teleoperation system's adaptive control structure based on the position error structure is shown in Figure 2. The control structure connects the position and velocity signals of the master-slave robot's end arm through forward and reverses channels to form a network. In this network, the desired position and speed signals of the master robot are the end position and speed signals of the slave robot actuator through the reverse channel. The desired position and speed signals of the slave robot are the end position, and speed signals of the main robot actuator passing through the forward channel, that is:


(26) $$\begin{array}{l} \{\begin{array}{c} x_{s}^{d}\left(t\right)=x_{m}\left(t-T_{m}\right)\\ x_{m}^{d}\left(t\right)=x_{s}\left(t-T_{s}\right) \end{array},\;\{\begin{array}{c} {\dot{x}}_{s}^{d}\left(t\right)={\dot{x}}_{m}\left(t-T_{m}\right)\\ {\dot{x}}_{m}^{d}\left(t\right)={\dot{x}}_{s}\left(t-T_{s}\right) \end{array} \end{array}$$

According to the self-adaptive control structure block diagram of the teleoperation system based on the position error structure, the robot's end position's tracking error at the master end and the slave end can be obtained. Thus, the end position's tracking error is obtained through the relationship between the end position of the master robot and the slave robot and the joint angle. That is, the position signal is converted into the joint signal, and the joint position tracking error of the master robot and the slave robot is obtained as follows:


(27) $$\begin{array}{l} e_{m}\left(t\right)=q_{m}\left(t\right)-q_{m}^{d}\left(t\right)=q_{m}\left(t\right)-q_{s}\left(t-T_{s}\right) \end{array}$$

(28) $$\begin{array}{l} e_{s}\left(t\right)=q_{s}\left(t\right)-q_{s}^{d}\left(t\right)=q_{s}\left(t\right)-q_{m}\left(t-T_{m}\right) \end{array}$$

The mathematical models of each module in the teleoperation system are unified into the joint space. They have substituted into the joint space mathematical models of the master robot and the slave robot.


(29) $$\begin{array}{l} {\hat{M}}_{m}\left(q_{m}\right){\ddot{q}}_{m}+{Ĉ}_{m}\left(q_{m},{\dot{q}}_{m}\right){\dot{q}}_{m}+{Ĝ}_{m}\left(q_{m}\right)={\hat{\tau }}_{m}=\\ Y_{zm}\left(q_{m},{\dot{q}}_{m},{\ddot{q}}_{m}\right){\hat{\theta }}_{zm} \end{array}$$

(30) $$\begin{array}{l} {\hat{M}}_{s}\left(q_{s}\right){\ddot{q}}_{s}+{Ĉ}_{s}\left(q_{s},{\dot{q}}_{s}\right){\dot{q}}_{s}+{Ĝ}_{s}\left(q_{s}\right)={\hat{\tau }}_{s}=\\ Y_{zs}\left(q_{s},{\dot{q}}_{s},{\ddot{q}}_{s}\right){\hat{\theta }}_{zs} \end{array}$$

Where, ${\hat{M}}_{m}\left(q_{m}\right),\;{Ĉ}_{m}\left(q_{m},{\dot{q}}_{m}\right),{Ĝ}_{m}$ are the estimated values of $M_{m}\left(q_{m}\right),C_{m}\left(q_{m},{\dot{q}}_{m}\right),G_{m}.$ ${\hat{M}}_{s}\left(q_{s}\right),{Ĉ}_{s}\left(q_{s},{\dot{q}}_{s}\right),{Ĝ}_{s}$ are the estimated values of unknown constant parameter vectors of the master robot and the slave robot. $Y_{z}\left(q_{i},{\dot{q}}_{i},{\ddot{q}}_{i}\right)$ are the dynamic regression function matrix of the known variables of the master and slave robot joints.

It can be seen from formula (28) and formula (29) that the dynamic uncertainty parameter vector θ_*zm*_ obtained by a linear transformation of the master robot dynamic combination model is composed of the dynamic uncertain parameters of the master robot and the operator. Similarly, the dynamic uncertainty parameter vector obtained from the linear transformation of the robot dynamics combination model consists of the uncertain parameters of the slave robot and the environment dynamics form. In this way, we include the external force $f_{h}^{*}$ of the operator and the external force $f_{e}^{*}$ of the environment into the dynamic uncertainty vector θ_*zm*_, θ_*zs*_ of the system.

### Controller design and stability analysis of teleoperation system

The control objective of the teleoperation system based on the position error control structure in this section is to design an adaptive controller at the master end and an adaptive controller at the slave end for the master robot and the slave robot, respectively, to solve the problem that the dynamic parameters of the master robot and the slave robot are uncertain. The communication channel has a constant time delay, ensuring the system's stability and making the system have stable performance. It has good instantaneous characteristics and transparency, thus improving the tracking performance of the system (Liu et al., 2020).

Therefore, based on the position error control structure, two-sided adaptive controllers are designed for the master robot and the slave robot, respectively. The stability and position tracking of the teleoperation system's performance is analyzed using high calculus Lyapunov Krasovski functional.

#### Controller design

Now we start to design non-linear bilateral adaptive controllers. Firstly, to stabilize the motion of the master robot and the slave robot in the teleoperation system, *t* → ∞, when ${\dot{q}}_{i}\left(t\right)\to 0$. The following auxiliary variables need to be defined:


(31) $$\begin{array}{l} s_{m}\left(t\right)={\dot{q}}_{m}\left(t\right)+\lambda_{m}e_{m}\left(t\right) \end{array}$$

(32) $$\begin{array}{l} s_{s}\left(t\right)={\dot{q}}_{s}\left(t\right)+\lambda_{s}e_{s}\left(t\right) \end{array}$$

Where λ_*i*_ is the diagonal matrix of positive definite constant and represents the weight of tracking error.

Since the acceleration signal cannot be used in a teleoperation system, a regression matrix ${\bar{Y}}_{i}\left(q_{i},{\dot{q}}_{i},e_{i},{ė}_{i}\right)$ about tracking error is defined as follows:


(33) $$\begin{array}{l} M_{i}\left(q_{i}\right)\lambda_{i}{ė}_{i}+C_{i}\left(q_{i},{\dot{q}}_{i}\right)\lambda_{i}e_{i}+G_{i}\left(q_{i}\right)={\bar{Y}}_{i}\left(q_{i},{\dot{q}}_{i},e_{i},{ė}_{i}\right)\theta_{zi} \end{array}$$

Formula (33) shows that when *e*_*i*_ → 0, there were ${Ȳ}_{i}\left(q_{i},{\dot{q}}_{i},e_{i},{ė}_{i}\right)\theta_{zi}\to G_{i}\left(q_{i}\right)$, *i* = *m, s*.

Thus, the adaptive controller of the master robot and the adaptive controller of the slave robot is designed as:


(34) $$\begin{array}{l} \tau_{m}=-{\bar{Y}}_{m}\left(q_{m},{\dot{q}}_{m},e_{m},{ė}_{m}\right){\hat{\theta }}_{zm}-k_{m}s_{m}-\beta_{m}{ė}_{m} \end{array}$$

(35) $$\begin{array}{l} \tau_{s}=-{\bar{Y}}_{s}\left(q_{s},{\dot{q}}_{s},e_{s},{ė}_{s}\right){\hat{\theta }}_{zs}-k_{s}s_{s}-\beta_{s}{ė}_{s} \end{array}$$

Where, for convenience, here *i* = *m, s*, *k*_*i*_ is a positive definite constant diagonal matrix, which represents the proportional gain. The term ${\bar{Y}}_{i}\left(q_{i},{\dot{q}}_{i},e_{i},{ė}_{i}\right){\hat{\theta }}_{i}$ is a model-based term, that is used to compensate for the error. ${\hat{\theta }}_{i}$ is the estimated value of θ_*i*_. The proportion term for auxiliary variables *s*_*i*_ is the additional term of error compensation. The other term β_*i*_ė_*i*_ is used to eliminate the additional energy dissipation caused by a time delay, where β_*i*_ is the diagonal matrix of positive definite constant, representing the dissipation coefficient.

By substituting formula (34) and formula (35) into formula (14) and formula (15), the following results are obtained:


(36) $$\begin{array}{l} M_{m}\left(q_{m}\right){ṡ}_{m}+C_{m}\left(q_{m},{\dot{q}}_{m}\right)s_{m}+2G_{m}\left(q_{m}\right)=\\ {Ȳ}_{m}\Delta \theta_{zm}-k_{m}s_{m}-\beta_{m}{ė}_{m} \end{array}$$

(37) $$\begin{array}{l} M_{m}\left(q_{m}\right){ṡ}_{m}+C_{m}\left(q_{m},{\dot{q}}_{m}\right)s_{m}+2G_{m}\left(q_{m}\right)=\\ {\bar{Y}}_{m}\Delta \theta_{zs}-k_{m}s_{m}-\beta_{m}{ė}_{m} \end{array}$$

Among them, Δθ_*zm*_, Δθ_*zs*_, respectively represents the estimated error of θ_*zm*_, θ_*zs*_, which satisfies $\Delta \theta_{zi}=\theta_{zi}-\;{\hat{\theta }}_{zi}$.

Usually, the adaptive law proposed by Slotine and Li (1991) is directly selected:


(38) $$\begin{array}{l} {\dot{\hat{\theta }}}_{zi}=-\Gamma_{i}^{-1}{\bar{Y}}_{i}^{T}s_{i} \end{array}$$

Among them, *i* = *m, s*, Γ_*i*_ is the positive definite diagonal constant matrix gain.

The above estimation method has been widely used in adaptive control applications. However, Slotine and Li (1991) also pointed out that if the reference signal does not meet the persistent excitation conditions, the above estimation accuracy cannot be guaranteed. Therefore, to improve the adaptive estimation value's accuracy and convergence rate, the above formula's estimation method is improved using the input torque estimation error. The adaptive estimation law of uncertain parameters θ_*zi*_ is defined as follows:


(39) $$\begin{array}{l} \{\begin{array}{c} {\dot{\hat{\theta }}}_{zi}=-\Gamma_{i}^{-1}\left(t\right)\left({\bar{Y}}_{i}^{T}s_{i}+\left(\xi_{1}+\xi_{2}\right)z_{i}+\xi_{1}Y_{i}^{T}\varepsilon_{i}\right)\\ \begin{array}{c} {\dot{z}}_{i}\left(t\right)=-\eta z_{i}\left(t\right)+\mu Y_{zi}^{T}\varepsilon_{i}-p_{i}\left(t\right){\dot{\hat{\theta }}}_{zi}\left(t\right) \end{array} \end{array} \end{array}$$

Among them, for the sake of convenience *i* = *m, s*, $\varepsilon_{i}=Y_{zi}\theta_{zi}-Y_{zi}{\hat{\theta }}_{zi}=Y_{zi}\Delta \theta_{zi}$ denotes the estimation error of calculating input torque used for correcting the estimated value. Here Δθ_*i*_ represents the estimation error. Γ_*i*_(*t*) = *diag*(γ_1_(*t*), ···, γ_*p*_(*t*)) is the adaptive gain matrix, satisfying Γ_*i*_(*t*) > 0, ${\dot{\Gamma }}_{i}\left(t\right)\leq 0$, where $\gamma_{n}\left(t\right)=a_{n}e^{-\int_{0}^{t}f_{n}\left(\upsilon \right)d\upsilon }+b_{n}$. *a*_*n*_. *b*_*n*_ are the constants, and *f*_*n*_(υ) ≥ 0, *n* = {1, ···, *p*}. *p*_*i*_(*t*) is the low-pass filtered signal of $Y_{zi}^{T}Y_{zi}$ which satisfies the formula $p_{i}\left(t\right)=\mu \int_{0}^{t}e^{-\eta \left(t-\sigma \right)}Y_{zi}^{T}\left(\sigma \right)Y_{zi}\left(\sigma \right)d\sigma$, where μ and η are constant and ξ_1_ and ξ_2_ are constant too. According to the robust control technique in Nuño et al. (2010), the coefficients ξ_1_ can be defined as:


(40) $$\begin{array}{l} \xi_{1}=\alpha \frac{\left\|{Ȳ}_{i}^{T}s_{i}\right\|}{{\lambda \left(Y_{zi}^{T}Y_{zi}\right)}_{\min}} \end{array}$$

where α, δ is the constant. ${\lambda \left(Y_{zi}^{T}Y_{zi}\right)}_{\min}$ is the minimum eigenvalue of $Y_{zi}^{T}Y_{zi}$.

#### Analysis of system stability and tracking performance

In this paper, the stability of closed-loop systems with time delay is described in formulas (14) and (15), adaptive bilateral control laws in formula (36) and formula (37), and adaptive laws shown in formula (39) are analyzed. The joint position tracking error ė_*m*_, ė_*s*_ and the estimation error of the adaptive law Δθ_*zm*_, Δθ_*zs*_ of the teleoperation system are bounded. The velocity ${\dot{q}}_{m},{\dot{q}}_{s}$ and tracking error ė_*m*_, ė_*s*_ converge to zero.:

For a teleoperation robot system, a high calculus Lyapunov Krasovski candidate function *V*(*t*) is defined:


(41) $$\begin{array}{l} V\left(t\right)=V_{1}\left(t\right)+V_{2}\left(t\right)+V_{3}\left(t\right)+V_{4}\left(t\right) \end{array}$$

Where, $V_{1}\left(t\right)=\frac{1}{2}\sum_{i=m,s}s_{i}^{T}M_{i}s_{i}$, $V_{2}\left(t\right)=\frac{1}{2}\sum_{i=m,s}\Delta \theta_{zi}^{T}\Gamma_{i}\left(t\right)\Delta \theta_{zi}$, $V_{3}\left(t\right)=\frac{1}{2}\underset{i=m,s}{\sum }e_{i}^{T}\lambda^{T}k_{i}\beta_{i}e_{i}$, and $V_{4}\left(t\right)=\frac{1}{2}\underset{i=m,s}{\sum }\int_{t-\Delta T}^{t}{\dot{q}}_{i}^{T}\left(\sigma \right)k_{i}\beta \dot{q}\left(\sigma \right)d\sigma$, for the sake of convenience, *i* = *m, s*.

From property (4), we know that *M*_*m*_(*q*_*m*_), *M*_*s*_(*q*_*s*_) are symmetric positive definite matrices, Γ_*i*_(*t*), *k*_*i*_, β_*i*_ are positive definite matrices. Therefore, *V*(*t*) is also positive definite matrices.

By deriving the two sides of the candidate Lyapunov function formula (41), the following results are obtained:


(42) $$\begin{array}{l} \dot{V}\left(t\right)={\dot{V}}_{1}\left(t\right)+{\dot{V}}_{2}\left(t\right)+{\dot{V}}_{3}\left(t\right)+{\dot{V}}_{4}\left(t\right)\\ \dot{V}\left(t\right)=\frac{1}{2}\underset{i=m,s}{\sum }s_{i}^{T}{Ṁ}_{i}s_{i}+\underset{i=m,s}{\sum }s_{i}^{T}M_{i}{ṡ}_{i}+\\ \frac{1}{2}\underset{i=m,s}{\sum }\Delta \theta_{zi}^{T}{\dot{\Gamma }}_{i}\left(t\right)\Delta \theta_{zi}+\underset{i=m,s}{\sum }\Delta \theta_{zi}^{T}\Gamma_{i}\left(t\right)\Delta {\dot{\theta }}_{zi}\\ +\underset{i=m,s}{\sum }e_{i}^{T}\lambda^{T}k_{i}\beta_{i}{ė}_{i}+\\ \frac{1}{2}\underset{i=m,s}{\sum }\left({\dot{q}}_{i}^{T}\left(t\right)k_{i}\beta \dot{q}\left(t\right)-{\dot{q}}_{i}^{T}\left(t-\Delta T\right)k_{i}\beta \dot{q}\left(t-\Delta T\right)\right) \end{array}$$

According to property (5):


(43) $$\begin{array}{l} \frac{1}{2}{s_{i}}^{T}{Ṁ}_{i}\left(q\right)s_{i}={s_{i}}^{T}\left(C_{i}\left(q,\dot{q}\right)-B_{i}\right){s_{i}}^{T} \end{array}$$

Because $\Delta \theta_{zi}=\theta_{zi}-{\hat{\theta }}_{zi}$, so there are:


(44) $$\begin{array}{l} \Delta {\dot{\theta }}_{zi}=-{\dot{\hat{\theta }}}_{zi} \end{array}$$

By substituting formulas (43) and (44) into formula (42), the results are as follows:


(45) $$\begin{array}{l} \;\dot{V}\left(t\right)=-\sum_{i=m,s}(s_{i}^{T}\left(k_{i}+B_{i}\right)s_{i}+\frac{1}{2}{\dot{e}}_{i}^{T}k_{i}\beta_{i}{\dot{e}}_{i}\\ +\Delta \theta_{zi}^{T}\left(\left(\xi_{1}+\xi_{2}\right)p_{i}+\xi_{1}Y_{zi}^{T}Y_{zi}\right)\Delta \theta_{zi}-\frac{1}{2}\Delta \theta_{zi}^{T}{\dot{\Gamma }}_{i}\Delta \theta_{zi}) \end{array}$$

From the previous definition, Γ_*i*_(*t*) > 0 and ${\dot{\Gamma }}_{i}\left(t\right)\leq 0$ are negative semidefinite, then


(46) $$\begin{array}{l} \dot{V}\left(t\right)\leq -\sum_{i=m,s}(s_{i}^{T}\left(k_{i}+B_{i}\right)s_{i}+\frac{1}{2}{\dot{e}}_{i}^{T}k_{i}\beta_{i}{\dot{e}}_{i}+\\ \Delta \theta_{zi}^{T}\left(\left(\xi_{1}+\xi_{2}\right)p_{i}+\xi_{1}Y_{zi}^{T}Y_{zi}\right)\Delta \theta_{zi}) \end{array}$$

Where, *B*_*i*_, *k*_*i*_, β_*i*_ is the positive definite diagonal constant, $\left(\xi_{1}+\xi_{2}\right)p_{i}+\xi_{1}Y_{zi}^{T}Y_{zi}$ is semi-positive definite, therefore $\dot{V}\left(t\right)\leq \;0$.

Therefore, *V*(*t*) ≥ 0 while $\dot{V}\left(t\right)\leq 0$. The auxiliary variables *s*_*i*_ of the system, the tracking error *e*_*i*_, and the estimation error Δθ_*zi*_ of the adaptive law are bounded. Then we use the theorem of Barbalat to know that the $\dot{V}\left(t\right)$ asymptotic approach to 0, then at that time *t* → ∞, there are *s*_*i*_ → 0, ė_*i*_ → 0.

Thus, $\underset{t\to \infty }{\lim}\left(s_{m}+s_{s}\right)=0$, define *r*(*t*) = *x*_*m*_ + *x*_*s*_. We can get the following conclusions:


(47) $$\begin{array}{l} \underset{t\to \infty }{\lim}\left(ṙ\left(t\right)+\lambda \left(r\left(t\right)-r\left(t-\Delta T\right)\right)\right)=0 \end{array}$$

Laplace transform formula (47) into: $\underset{t\to \infty }{\lim}\left(sr\left(s\right)-r\left(0\right)+\lambda \left(r\left(s\right)-e^{-s\Delta T}r\left(s\right)\right)\right)=\;0$,

It can be inferred that $\underset{t\to \infty }{\lim}s^{2}r\left(s\right)=0$. that is ẋ_*m*_ + ẋ_*s*_ → 0 when *t* → ∞.

Similarly, define *r*(*t*) = *x*_*m*_ − *x*_*s*_, get ẋ_*m*_ − ẋ_*s*_ → 0 as *t* → ∞. Therefore, it is proved that the velocity of the master robot and the slave robot approaches zero asymptotically, that is ẋ_*m*_, ẋ_*s*_ → 0, as *t* → ∞. Considering the auxiliary variables *s*_*i*_ = ẋ_*i*_ + λ_*i*_*e*_*i*_ → 0, the position tracking error converges to 0.

### Simulation verification

The master robot and the slave robot are simulated, respectively, in free motion and in contact with the operator and the environment to verify the above control algorithm's effectiveness. In these two cases, the position tracking and force tracking of the traditional adaptive control algorithm in Nuño et al. (2010) and the designed adaptive control method are compared.

**Figure 3:**
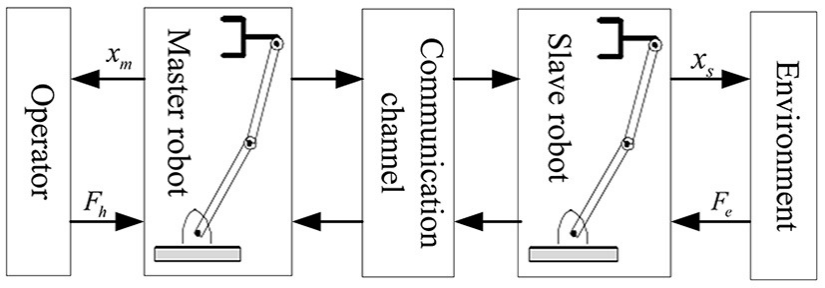
Schematic diagram of teleoperation system selected by simulation.

In this paper's simulation experiment, the teleoperation system structure is selected, as shown in Figure 3. The master and slave robots of the teleoperation system with time-delay force feedback are selected as the two degrees of freedom two-link rotary manipulator robots. Its dynamic mathematical model is formula (48) ~ formula (52). As shown in Figure 3, the kinematic Jacobian matrices of the master and slave robots of the closed-loop delay teleoperation system are:


(48) $$\begin{array}{l} M\left(q\right)\ddot{q}+C\left(q,\dot{q}\right)\dot{q}+G\left(q\right)=\tau \end{array}$$

Among which,


(49) $$\begin{array}{l} \tau =\left[\begin{array}{c} \tau_{1}\\ \tau_{2} \end{array}\right] \end{array}$$

(50) $$\begin{array}{l} M\left(q\right)=\left[\begin{array}{cc} p_{1}+p_{2}+2p_{3}\cos\left(q_{2}\right) & p_{2}+p_{3}\cos\left(q_{2}\right)\\ p_{2}+p_{3}\cos\left(q_{2}\right) & p_{2} \end{array}\right] \end{array}$$

(51) $$\begin{array}{l} C\left(q,\dot{q}\right)=\left[\begin{array}{cc} -p_{3}{\dot{q}}_{2}\cos\left(q_{2}\right) & -p_{3}\left({\dot{q}}_{1}+{\dot{q}}_{2}\right)\sin\left(q_{2}\right)\\ p_{3}{\dot{q}}_{1}\sin\left(q_{2}\right) & 0 \end{array}\right] \end{array}$$

(52) $$\begin{array}{l} G\left(q\right)=\left[\begin{array}{c} p_{4}g\cos\left(q_{1}\right)+p_{5}g\cos\left(q_{1}+q_{2}\right)\\ p_{5}g\cos\left(q_{1}+q_{2}\right) \end{array}\right] \end{array}$$

Here, g is the acceleration of gravity $p_{1}=m_{1}l_{c1}^{2}+m_{2}l_{1}^{2}+I_{1}$, $p_{2}=m_{2}l_{c2}^{2}+I_{2}$, *p*_3_ = *m*_*c*_*l*_1_*l*_*c*2_, *p*_4_ = *m*_1_*l*_*c*2_ + *m*_2_*l*_1_, and *p*_5_ = *m*_2_*l*_*c*2_.


(53) $$\begin{array}{l} J\left(q\right)=\left[\begin{array}{cc} -l_{1}\sin\left(q_{1}\right)-l_{2}\sin\left(q_{1}+q_{2}\right) & -l_{2}\sin\left(q_{1}+q_{2}\right)\\ l_{1}\cos\left(q_{1}\right)+l_{2}\cos\left(q_{1}+q_{2}\right) & \cos\left(q_{1}+q_{2}\right) \end{array}\right] \end{array}$$

The end effector position of the master and slave robots in the system is expressed in the plane rectangular coordinate system as follows:


(54) $$\begin{array}{l} {\left[x,y\right]}^{T}=h\left(q\right)=\\ \left[l_{1}\cos\left(q_{1}\right)+l_{2}\cos\left(q_{1}+q_{2}\right),l_{1}\sin\left(q_{1}\right)+l_{2}\sin\left(q_{1}+q_{2}\right)\right] \end{array}$$

In the simulation verification, the dynamic parameters of the master robot and the slave robot are shown in Table 1.

**Table 1:** Physical parameters of master-slave robot double joint manipulator.

**Name**	**Symbol**	**Value**	**Unit**
Mass of rod 1	*m*_1_	1	kg
Length of rod 1	*l*_1_	1	m
Distance from the centroid of rod 1 to joint 0	*l*_*c*1_	1/2	m
Moment of inertia of rod 1	*I*_1_	1/12	*kg* × *m*^2^
Mass of rod 2	*m*_2_	3	kg
Length of rod 2	*l*_2_	2	m
Distance from the centroid of rod 2 to joint 1	*l*_*c*2_	1	m
Moment of inertia of rod 2	*I*_2_	2/5	*kg* × *m*^2^

#### Simulation of free motion

Firstly, in the teleoperation system based on the position error structure shown in Figure 2, the effectiveness of the adaptive control method proposed in this chapter is verified by simulation in the case of the free motion of the master robot and the slave robot. There is no contact force in the process of the master robot and the robot, so the expression formula is *F*_*h*_ = 0 and *F*_*e*_ = 0.

The simulation experiment is carried out by using the MATLAB Simulink tool. The experiment proves that the bilateral teleoperation system with the dynamic model of formula (14) and formula (15) is stable under the control of adaptive controller type formula (34) and formula (35) and adaptive law formula (39). The position tracking error is bounded and can converge to near 0.

In the experiment, it is assumed that the dynamic parameters of the master robot and the slave robot are unknown. Let the dynamic parameter vectors of the master robot and slave robot be:


(55) $$\begin{array}{l} \theta_{m}=\theta_{s}={\left[\begin{array}{ccc} o & \nu & \kappa \end{array}\right]}^{T} \end{array}$$

Here $=m_{1}l_{c1}^{2}+m_{2}l_{1}^{2}+I_{1}+m_{2}l_{c2}^{2}+I_{2}$, $\nu =m_{2}l_{c2}^{2}+I_{2}$, and κ = *m*_*c*_*l*_1_*l*_*c*2_. The initial parameters of the master robot and the slave robot of the teleoperation system are set as initial position parameters *x*_*m*_(0) = [0.184, 2.6389] and *x*_*s*_(0) = [3, 0]. The initial dynamic uncertainty parameter vector is $\theta_{m}\left(0\right)=\theta_{s}\left(0\right)={\left[4.1,1.9,1.7\right]}^{T}$. The gain parameters of the master controller and slave controller are set *k*_*m*_ = *diag*(30, 30) and *k*_*s*_ = *diag*(30, 30), dissipation coefficient β_*m*_ = β_*s*_ = *diag*(0.8, 0.6), and λ_*m*_ = λ_*s*_ = *diag*(5, 5). The adaptive gain matrix of master adaptive law and slave adaptive law is as follows:


(56) $$\begin{array}{l} \Gamma_{m}\left(t\right)=\Gamma_{s}\left(t\right)=diag\left(e^{-t}+1,e^{-t}+1,e^{-t}+1\right) \end{array}$$

**Figure 4:**
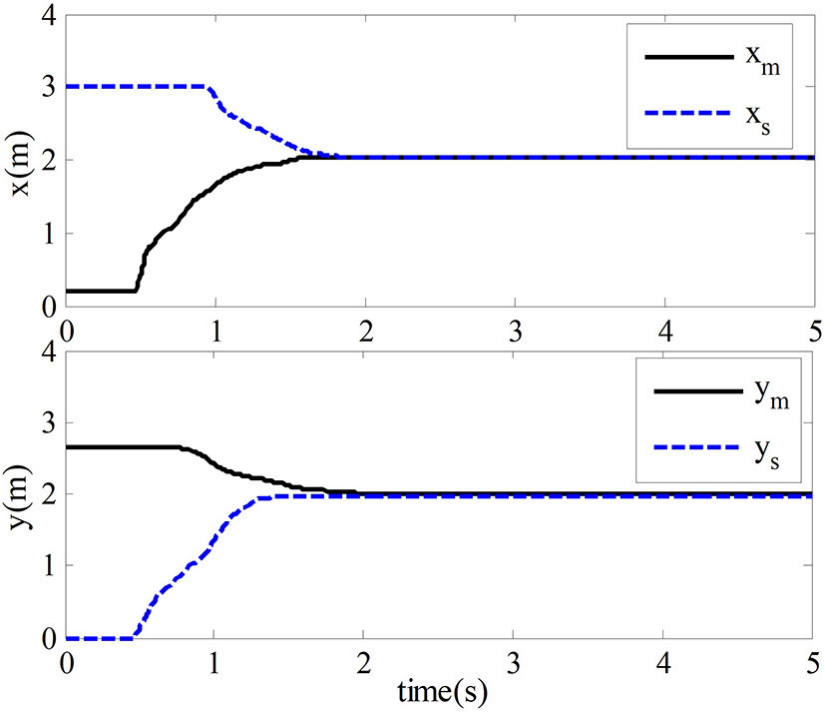
Trajectory map of end position tracking.

**Figure 5:**
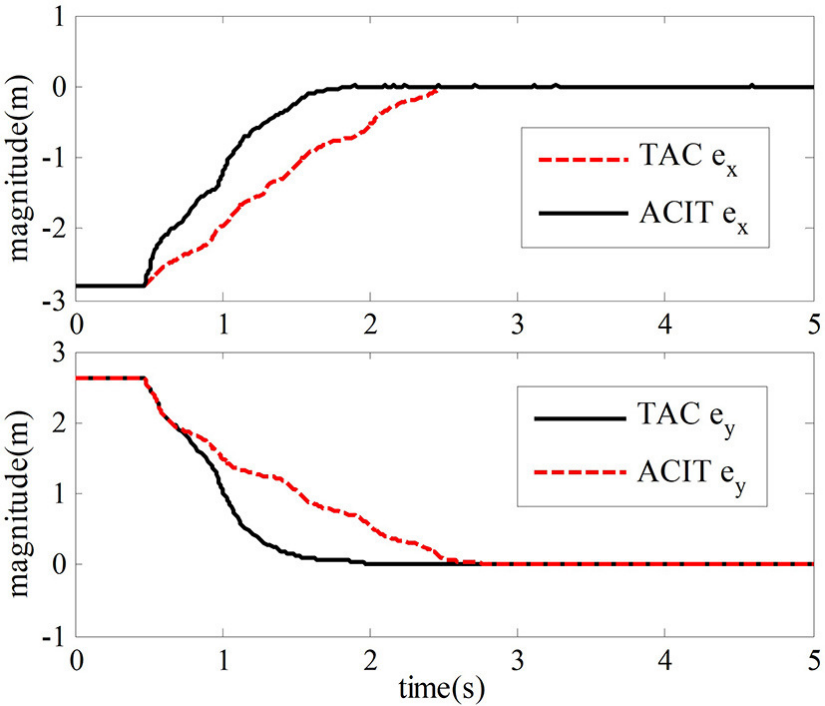
Tracking error of the end position of the robot.

And the constant parameters of the adaptive law and the slave adaptive law are set to α_*m*_ = α_*s*_ = 1, δ_*m*_ = δ_*s*_ = 1, ξ_2*m*_ = ξ_2*s*_ = 0.8, μ_*m*_ = μ_*s*_ = 1, and η_*m*_ = η_*s*_ = 1. Acceleration of gravity *g* = 9.81. In the teleoperation system, the forward channel delay and the reverse channel delay are *T*_*m*_ = *T*_*s*_ = 0.5 *s*. The simulation results are shown in Figures 4, 5.

The traditional adaptive two-sided control method of the time-delay teleoperation system in Nuño et al. (2010) to illustrate the advantages of the two-sided controller are compared and analyzed. The control law is as follows:


(57) $$\begin{array}{l} \tau_{i}=Y_{id}{\hat{\theta }}_{id}-K_{i}\left({ė}_{i}+\alpha_{i}e_{i}\right) \end{array}$$

The adaptive law is as follows:


(58) $$\begin{array}{l} {\hat{\theta }}_{id}=-L_{id}Y_{id}^{T}r_{i} \end{array}$$

Where *r*_*i*_ = ė_*i*_ + α_*i*_*e*_*i*_, α_*i*_, *K*_*i*_, *L*_*id*_ are constant diagonal matrix. In the simulation experiment, after repeated debugging, we can analyze and set the parameters of bilateral controller and adaptive law as α_*m*_ = α_*s*_ = 0.25, *K*_*m*_ = *K*_*s*_ = *diag*(100, 100), and *L*_*md*_ = *L*_*sd*_ = *diag*(40, 40).

#### Simulation with contact

In the case of contact motion, the operator contacts the master robot. From the contact between the robot and the environment, the effectiveness of the adaptive bilateral controller scheme designed in this chapter for the time-delay force feedback teleoperation system is verified.

This section also uses MATLAB SIMULINK for simulation verification. In the simulation teleoperation system and the environment space model is set. The environmental dynamic parameters are set as *m*_*e*_ = 0.1 *kg*, where the mass coefficient *m*_*e*_ = 0.1 *kg*, damping coefficient *b*_*e*_ = 20 *Ns*/*m*, elastic coefficient *b*_*e*_ = 20*Ns*/*m*, and external environmental force $f_{e}^{*}={\left[\begin{array}{cc} 0 & 0 \end{array}\right]}^{T}$ are set to simulate the passive environment. According to the operator model, the external force of the operator is set as $f_{h}^{*}={\left[\begin{array}{cc} f_{h1}^{*} & 0 \end{array}\right]}^{T}={\left[\begin{array}{cc} 25\cos\left(\pi t\right) & 0 \end{array}\right]}^{T}$. Moreover, its dynamic parameters are set as: *M*_*h*_ = *m*_*h*_*I*, *B*_*h*_ = *b*_*h*_*I*, and *K*_*h*_ = *k*_*h*_*I*, where the mass coefficient *m*_*h*_ = 0.2 *kg*, damping coefficient *b*_*h*_ = 50 *Ns*/*m*, and elastic coefficient are set to *k*_*h*_ = 100Ñ/*m*.

In the experiment, it is considered that the dynamic parameters of the master robot and the slave robot are unknown, and the bilateral adaptive controller can solve this problem. According to properties (6), the dynamic models of the master robot and the slave robot are transformed linearly. The dynamic uncertain parameter vectors are obtained as follows:


(59) $$\begin{array}{l} \theta_{m}=[l_{c2}^{2}m_{2}+l_{2}^{2}M_{h},l_{1}l_{c2}m_{2},l_{1}l_{2}m_{h},l_{1}l_{2}b_{h},l_{2}^{2}b_{h},l_{1}l_{2}k_{h},\\ \;{l_{1}^{2}m_{2}+I_{1}+I_{2}+l_{c1}^{2}m_{1}+l_{1}^{2}m_{h},l_{1}^{2}b_{h},f_{h1}^{*}l_{1},f_{h1}^{*}l_{2}]}^{T} \end{array}$$

(60) $$\begin{array}{l} \theta_{s}=[l_{c2}^{2}m_{2}+l_{2}^{2}M_{e},l_{1}l_{c2}m_{2},l_{1}l_{2}m_{e},l_{1}l_{2}b_{e},l_{2}^{2}b_{e},l_{1}l_{2}k_{e},\\ {\;l_{1}^{2}m_{2}+I_{1}+I_{2}+l_{c1}^{2}m_{1}+l_{1}^{2}m_{e},l_{1}^{2}b_{e}]}^{T} \end{array}$$

By introducing the parameters in Table 1 into formula (59) and formula (60), the actual dynamic parameter vector can be calculated as follows:


$$\begin{array}{l} \theta_{m}=[3.8,3,0.4,100,200,200,3.933,50,25\\ \;{\left(1-\cos\left(\pi t\right)\right),50\left(1-\cos\left(\pi t\right)\right)]}^{T}\;\\ \theta_{s}={\left[3.4,3,0.2,100,200,200,3.833,50\right]}^{T}\; \end{array}$$

In the case of contact, the initial positions of the master robot and the slave robot of the teleoperation system are the same. That is, the initial position parameters *x*_*m*_(0) = [0.184, 2.6389] and *x*_*s*_(0) = [0.184, 2.6389]. The parameters of the master robot and the slave robot are shown in Table 1. Acceleration of gravity *g* = 9.81. Set the initial dynamic parameter vector as follows:


$$\begin{array}{l} \theta_{m}\left(0\right)={\left[1.9,1.7,0.5,90,190,180,2.0,40,20,45\right]}^{T}\;\\ \theta_{s}\left(0\right)={\left[1.9,1.7,0.1,90,190,190,1.9,40\right]}^{T}\; \end{array}$$

After repeated debugging, the design parameters of the master controller and slave controller formula (33) and formula (34) are set as follows *k*_*m*_ = *diag*(50, 50) and *k*_*s*_ = *diag*(50, 50). gain parameter is β_*m*_ = β_*s*_ = *diag*(0.8, 0.6) and λ_*m*_ = λ_*s*_ = *diag*(5, 5). Dissipation coefficient is β_*m*_ = β_*s*_ = *diag*(0.8, 0.6). The adaptive law of the master robot and the slave robot is formula (3-14). After repeated debugging, the adaptive gain matrix of the master robot and the slave robot is: $\Gamma_{m}\left(t\right)=\left(e^{-t}+2\right)I_{8\times 8}$ and $\Gamma_{s}\left(t\right)=\left(e^{-t}+2\right)I_{6\times 6}$. The constant parameters of the adaptive law and the slave adaptive law are set to α_*m*_ = α_*s*_ = 1, δ_*m*_ = δ_*s*_ = 1, ξ_2*m*_ = ξ_2*s*_ = 0.8, μ_*m*_ = μ_*s*_ = 1, and η_*m*_ = η_*s*_ = 1. In the teleoperation system, the forward channel delay and the reverse channel delay are *T*_*m*_ = *T*_*s*_ = 0.5*s*. The experimental results are shown in **Figure 7**.

The adaptive two-sided controller described in formula (57) and adaptive law described in formula (58) in Nuño et al. (2010) are compared under the same environmental conditions to illustrate the advantages of the two-sided controller. The parameters of the controller in Nuño et al. (2010) are set to α_*m*_ = α_*s*_ = 5, *K*_*m*_ = *K*_*s*_ = 200*I*, *L*_*md*_ = *L*_*sd*_ = 40*I*.

## Results and findings

### Simulation results of free motion

This section verifies the proposed adaptive control method's effectiveness while the master robot and slave robot are in free motion. The proposed method is compared with the method mentioned in Nuño et al. (2010), which could not be fully discussed and explained in this paper. In the simulation experiment, when the teleoperation system moves freely, the master robot and the slave robot are not affected by external forces. Their position tracking trajectory is shown in Figure 4. We can tell from Figure 4 that the slave robot of the teleoperation system can track the position of the master robot within 1.8 s and ensure the stability of the closed-loop system with time-delay force feedback.

**Figure 6:**
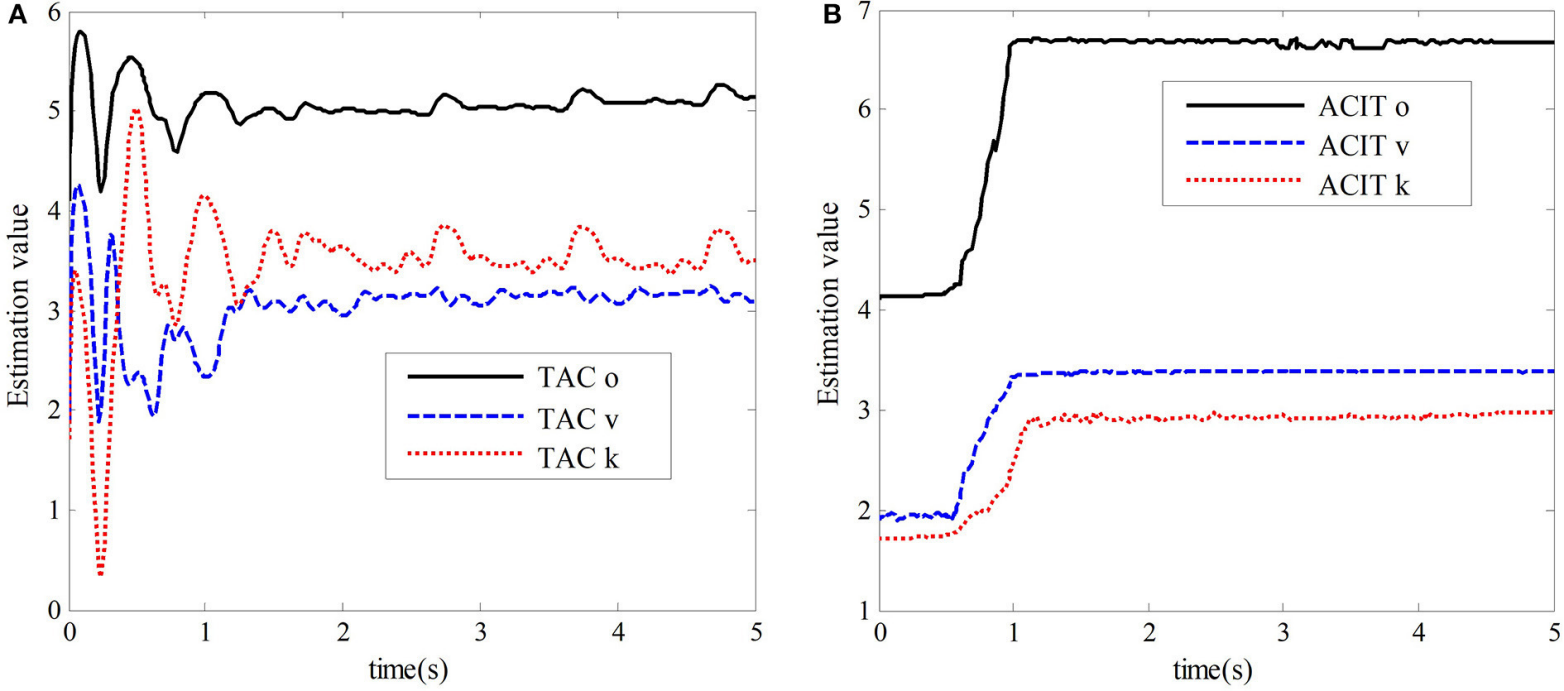
Estimated values of the dynamic parameter vector. **(A)** The estimated value of TAC. **(B)** The estimated value of ACIT.

The simulation results of the comparative experiment are shown in Figures 5, 6. In the figures, ACIT (adaptive bilateral control with improved tracking performance) represents the bilateral adaptive control method designed. TAC (traditional adaptive bilateral controller) represents the traditional bilateral adaptive control method in Nuño et al. (2010).

Figure 5 shows the end position tracking error curves of the master robot and the slave robot of the teleoperation system with time-delay force feedback under the two control methods. From this graph, we can observe that the time of position tracking error convergence to zero in the two-sided adaptive control method is about 1.8 s. In comparison, the traditional adaptive control method is about 2.8 s.

Figures 6A,B show the estimated values of the dynamic uncertain parameter vector of the slave robot of the teleoperation system under the traditional bilateral adaptive control method and the bilateral adaptive control method designed in this chapter, respectively. For the time-delay teleoperation system with uncertain dynamic parameters, the adaptive bilateral control method designed in this chapter can quickly converge to the real value in 0.5 s compared with the traditional two-sided adaptive control method in Nuño et al. (2010). However, in the traditional adaptive control method, it takes 1.8 s to converge to the region near the real value, which is unstable and fluctuates greatly. Its accuracy is relatively low.

#### Simulation results with contact

**Figure 7:**
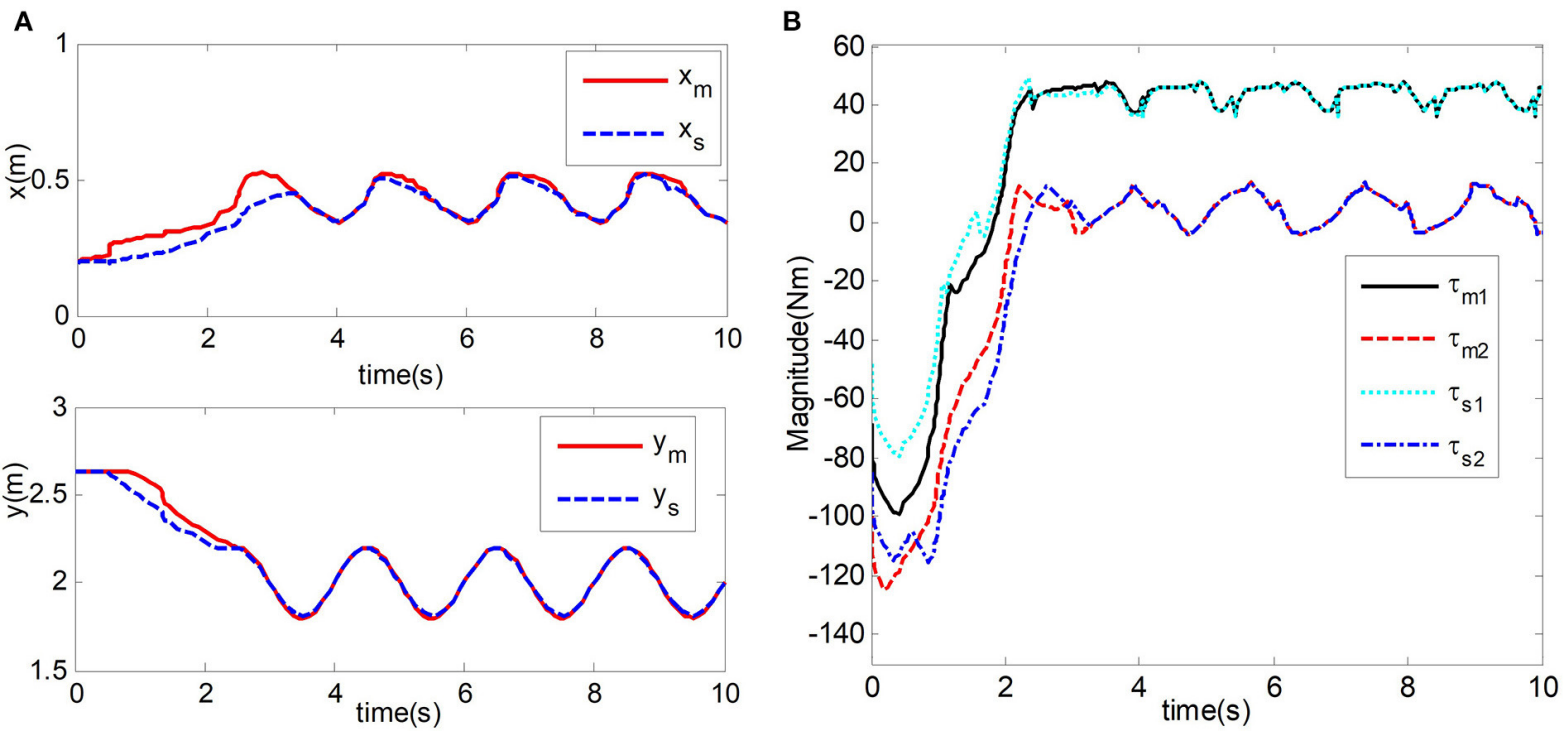
Simulation results. **(A)** End position trajectory of Master and slave robot. **(B)** Input torque of master and slave robot joints.

In the case of contact motion, the operator contacts the master robot. From the contact between the robot and the environment, the effectiveness of the adaptive bilateral controller scheme designed in this chapter for the time-delay force feedback teleoperation system is verified. The experimental results are shown in Figure 7.

Figure 7A shows the position tracking the trajectory of the mechanical end of the master robot and the slave robot of the teleoperation system. We can observe that the position curves of the master robot and the slave robot almost coincide in about 3.5 s. In Figure 7B, The input torques of joint 1 and joint 2 of the master robot and slave robot are displayed. The slave robot of the teleoperation system can track the position of the upper master robot under the contact condition, and the time-delay force feedback teleoperation system can maintain stability.

**Figure 8:**
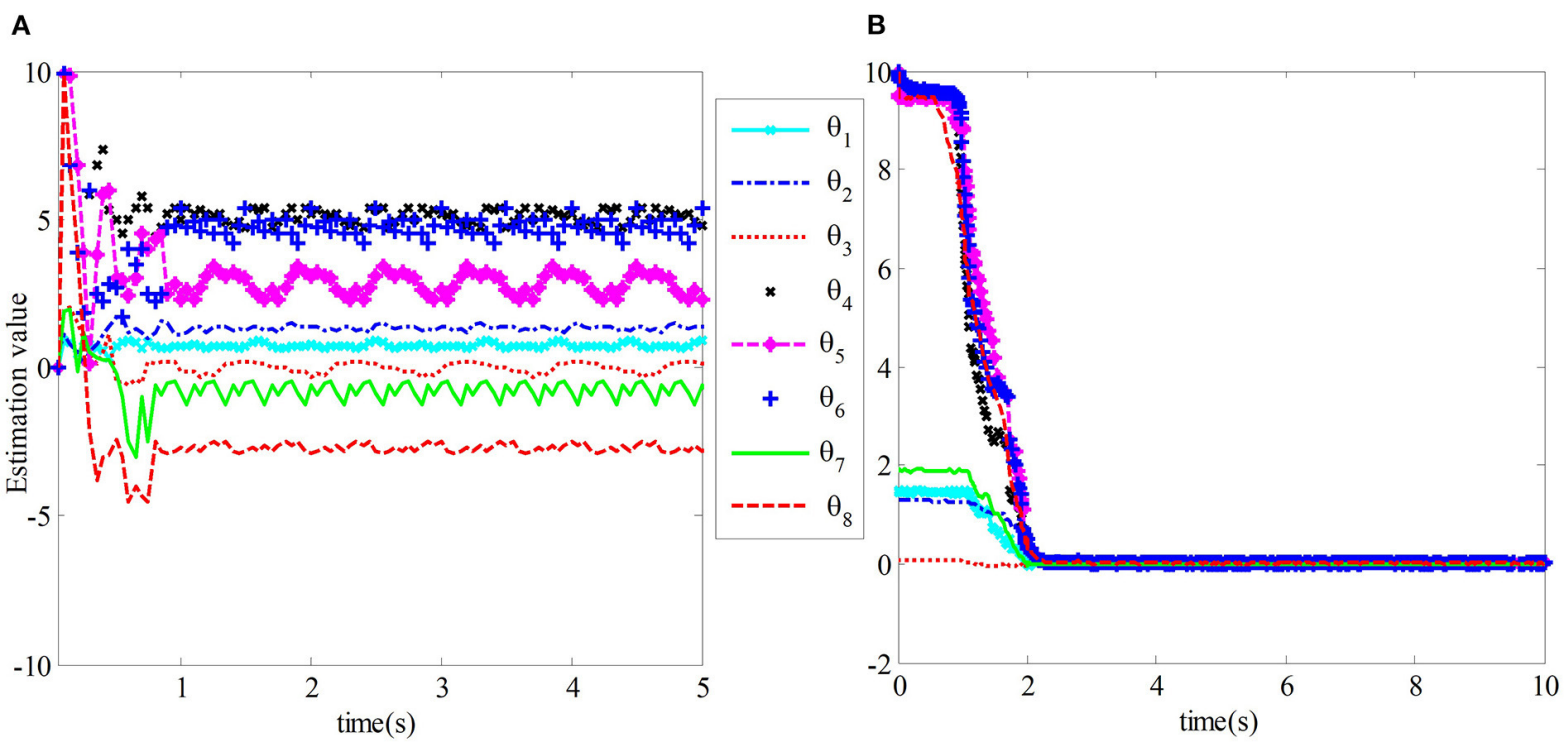
The estimation error of **θ**_**s**_ of the slave robot dynamic parameter vector. **(A)** The estimation error of TAC. **(B)** The estimation error of ACIT.

**Figure 9:**
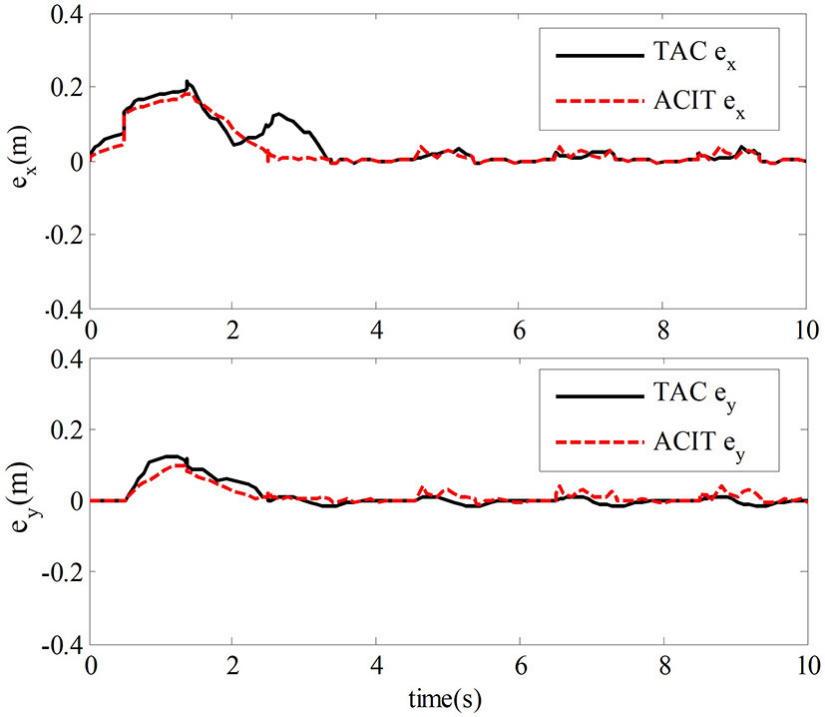
Position tracking error comparison of master-slave robot.

The comparison of experimental results shows that the estimation error of uncertain vector of dynamic parameters of the teleoperation system with time-delay force feedback under the two control methods and the tracking error curve of the master robot and the slave robot end position is shown in Figures 8, 9. In the figure, TAC is used to represent the method in Nuño et al. (2010). ACIT represents the method designed in this paper.

Figure 8A shows the estimation error of the uncertainty vector θ_*s*_ of the robot dynamic parameters for the teleoperation system under the traditional adaptive control method in Nuño et al. (2010). We can observe that the position tracking convergence speed of the teleoperation system under the traditional adaptive control method is slow, and the tracking error accuracy is not high. At the same time, its adaptive estimation converges slowly and fluctuates greatly, which will affect the control performance of the system in practical application. Figure 8B shows the estimated error of the teleoperation system from the uncertain vector θ_*s*_ of robot dynamic parameters under the control method designed in this paper. The estimated vector value from the uncertainty of robot dynamic parameters can quickly converge to the true value in 2 s.

Figure 9 compares the traditional adaptive control method and the control method designed in this chapter. We can observe that the adaptive control method in this paper has better tracking performance.

## Conclusions

Aiming at the uncertainty of dynamic parameters and time delay in the system, a bilateral adaptive control method based on PEB control structure is designed for a class of time-delay force feedback teleoperation systems without external interference and internal friction. The stability and tracking performance of the closed-loop constant time delay teleoperation system is analyzed by Lyapunov stability theory. Finally, the controller designed in this paper is successfully applied to the teleoperation system composed of a two-degree freedom rotating manipulator as a master robot and a slave robot. The advantages of this method are verified by simulation under the conditions of free motion and environmental forces and with contact and environmental forces, respectively. The control performance of this method is compared with that of the traditional adaptive bilateral control method for the time-delay teleoperation system, and the effectiveness of this method is analyzed by experiments. Finally, the method is proved to have good control performance.

The bilateral controller designed in this paper has the following advantages:

1. Based on the position error structure, the operator and environment's dynamic parameters can be included in the unknown vector of the whole system for estimation to avoid the influence of uncertain parameters in the operator and environment model on the system.
2. The estimation error is used to compensate for the adaptive estimation law to improve the system's tracking performance to improve the adaptive estimation accuracy and convergence speed.
3. By compensating for the dissipation caused by a time delay, the controller's performance is improved, thus ensuring the system's stability and improving the system's tracking performance.

There are still many possible improvements and experiments that could be performed based on this paper. For example, for more complex cases, such as non-linear models and time-varying cases, the control of teleoperation systems needs to be further studied. Besides, This paper only analyzes and verifies the control method of the time-delay force feedback teleoperation system in this article from two aspects of theory and simulation. There may be some problems that have not been considered in the actual system. Therefore, in future research work, the above method should be applied to the real teleoperation system for verification and improvement, so as to make it more practical.

## Data availability statement

The original contributions presented in the study are included in the article/supplementary material, further inquiries can be directed to the corresponding author/s.

## Author contributions

WZ and BY contributed to the conceptualization. ShL contributed to the methodology of this work and supervision. SiL, YB, and LY completed the writing of the original manuscript. XZ completed technical work such as model designing. XZ, SiL, and YB contributed to formal analysis and data curation. SiL, WZ, and LY carried out writing review and editing. WZ contributed to funding acquisition. SiL, YB, XZ, BY, ShL, LY, and WZ read and agreed to the published version of the manuscript. All authors contributed to the article and approved the submitted version.

## Funding

This work was jointly supported by the Sichuan Science and Technology Program (2021YFQ0003).

## Conflict of interest

The authors declare that the research was conducted in the absence of any commercial or financial relationships that could be construed as a potential conflict of interest.

## Publisher's note

All claims expressed in this article are solely those of the authors and do not necessarily represent those of their affiliated organizations, or those of the publisher, the editors and the reviewers. Any product that may be evaluated in this article, or claim that may be made by its manufacturer, is not guaranteed or endorsed by the publisher.
